# Comparison of Complication and Success Rates of ProGlide Closure Device in Patients Undergoing TAVI and Endovascular Aneurysm Repair

**DOI:** 10.1155/2018/2687862

**Published:** 2018-08-09

**Authors:** Gündüz Durmuş, Erdal Belen, Akif Bayyiğit, Mehmet Mustafa Can

**Affiliations:** ^1^Department of Cardiology, Haseki Education and Research Hospital, Istanbul, Turkey; ^2^Department of Internal Medicine, Okmeydanı Education and Research Hospital, Istanbul, Turkey

## Abstract

**Introduction:**

Usage of the Perclose ProGlide^®^ (PP: Abbott Laboratories, Chicago, IL, USA) closure device is becoming increasingly prevalent during percutaneous endovascular aortic repair (EVAR) and transcatheter aortic valve implantation (TAVI). The respective conditions treated via these procedures, abdominal aortic aneurysm and aortic valve stenosis, share risk factors but are two different physiopathological problems.

**Aim:**

Our aim was to compare the complication and success rates of PP closure device use in patients undergoing EVAR and TAVI.

**Materials and Methods:**

A total of 74 patients, including 58 undergoing TAVI and 16 undergoing EVAR, were analysed in our study.

**Results:**

Of the TAVI patients treated using the PP closure device, two (3.4%) had access to site-related bleeding complications and two (3.4%) experienced device failure. Of the EVAR patients who received the PP closure device, three (18.8%) had bleeding complications and three (18.8%) experienced device failure.

**Conclusion:**

Due to the underlying diffuse aortic wall pathology, the success rate of PP closure device use was lower and the complication rate of PP closure device was higher in the EVAR group versus the TAVI group.

## 1. Introduction

The application of percutaneous endovascular aortic repair (EVAR) instead of the classical surgical approach for aortic repair in patients with abdominal aortic aneurysm (AAA) has been found to reduce the length of hospital stay and increase patient comfort in addition to being noninferior in terms of outcomes [1–4]. Moreover, transcatheter aortic valve implantation (TAVI) has spread rapidly as a strategy to manage high-risk patients requiring aortic valve replacement due to aortic valve stenosis.

For this purpose, the common femoral artery is the most frequently used peripheral artery, and postprocedure hemostasis is often achieved by manual compression. However, manual compression can lead to the patient being unable to move for a period of time as well as back pain and local pain-induced vasovagal reaction during compression. Therefore, the use of local closure devices has entered into daily practice. Patients feel less pain, hemostasis is achieved faster, and patients are mobilized and discharged earlier following the use of closure devices as compared with compression [5–7].

The data for the complication and success rates of closure devices have accumulated rapidly. However, there are no data on the comparison of their use between in EVAR and TAVI patients, despite them being most commonly used in these individuals. Although the main pathology of aortic valve stenosis is on the valve, the main pathology of aortic aneurysm is inflammation and proteolytic degeneration in the aortic wall as well as thinning and dilatation in the whole wall, especially in the media layer. 

*Aim*. In light of this, the aim of the present study was to compare the complication and success rates of the Perclose ProGlide (PP: Abbott Laboratories, Chicago, IL, USA) closure device in these two different physiopathological conditions.

## 2. Materials and Methods

Patients who received the PP closure device during TAVI or EVAR procedures between 2015 and 2017 at our centre were retrospectively examined. Those who had excessive tortuosity in the iliac arteries, who demonstrated atherosclerotic stenosis greater than 50% or a femoral artery with a diameter of less than 6 mm, who had prosthetic arterial graft material in the intervention area, and/or who had undergone previous inguinal surgery, were not included in the study. The puncture site of all patients was evaluated by computed tomographic angiography prior to the procedure. The demographic characteristics and comorbidities of all patients were recorded. In all patients, venous blood samples were drawn and routine biochemical analyses were performed. The current study was approved by the institutional ethics committee and the investigation conformed to the principles of the Declaration of Helsinki.

Each PP closure device was implemented by the same trained and experienced operator. The procedures were performed under general anaesthesia. It was introduced between the iliac bifurcation and the inguinal ligament using the Seldinger technique for arterial puncture. Prior to the placement of the PP closure device, femoral angiography was performed to measure vessel diameter, calcification, and amount of tortuosity. Arterial access was obtained percutaneously at an oblique angle (45°). A small skin opening was made to permit PP advancement and the closure device was advanced over a 0.035 inch guidewire and deployed. Two sutures were placed in each arteriotomy by use of either two 6-French (Fr) PG devices sequentially deployed with opposite 30° rotation in a “crosshair” configuration. Intravenous heparin was administered at 1 mg/kg.

Device-related vascular complications are defined as access site bleeding and the occlusion, stenosis, and dissection of the artery used for access. The Bleeding Academic Research Consortium (BARC) recommendations were used as the bleeding classification measure in the present study [8]. Failure to suture, suture rupture, and/or breakage and inability to tighten the knot were defined as device failure.

### 2.1. Statistical Analysis

Statistical analyses were performed using the Statistical Package for the Social Sciences version 17.0 (IBM Corp., Armonk, NY, USA). Continuous variables were given in the format of mean ± standard deviation (if normal distribution) or median (interquartile range) (if not normal distribution), while categorical variables were given as percentages. The chi-squared test was used to compare the categorical variables between the groups. The Kolmogorov–Smirnov test was used to assess whether the variables were normally distributed. Student's t-test or Mann–Whitney U test was used to compare the continuous variables between the groups according to whether they were normally distributed or not. The results were evaluated within a 95% confidence interval and at a significance level of p < 0.05.

## 3. Results

A total of 74 patients, including 58 who underwent TAVI and 16 who underwent EVAR, were analysed in our study. The mean age of the patients was 74.8 years ± 8.4 years. Forty-four (59.5%) of the patients were male.

When demographic data and current comorbidities of the TAVI and EVAR patients who received PP closure devices were analysed, hypertension [14 (24.1%) vs. nine (56.3%), p = 0.030] and hyperlipidaemia [19 (32.8%) vs. 11 (68.8%), p = 0.009] were found to be more common in the EVAR patients. However, the mean age of the TAVI patients was higher than that of the EVAR patients [76.3 ± 8.3 years vs. 69.6 ± 6.9 years, p = 0.004] (Table 1). There was no significant difference between the groups in terms of biochemical parameters (Table 2).

Of the TAVI patients who received PP closure devices, two (3.4%) (including one patient with BARC class 2 and one patient with BARC class 3) had access site-related bleeding complications, while two (3.4%) experienced device failure. Of the EVAR patients who received PP closure devices, three (18.8%) (including one patient with BARC class 1, one patient with BARC class 2, and one patient with BARC class 3) had bleeding complications, while three (18.8%) demonstrated device failure. Complication frequency and device failure were significantly higher in the EVAR group versus in the TAVI group. Moreover, the number of PP closure devices for vascular occlusions per patient was significantly higher in the EVAR group than in the TAVI group (Table 3).

## 4. Discussion

In this study, we found that PP closure device-related complications and device failure were more common in EVAR patients than in TAVI patients.

Vascular closure devices are classified as either passive or active devices. Passive devices include haemostatic pads (e.g., Chitoseal™ from Abbott Laboratories, Chicago, IL, USA and Neptune Pad from Biotronik, Berlin, Germany) and compression devices (e.g., FemoStop^®^). Active devices include those that are collagen-based (e.g., VasoSeal and Angio-Seal™), clip-based (e.g., StarClose from Abbott Laboratories, Chicago, IL, USA), or suture-based (e.g., PP; ProStar from Abbott Laboratories, Chicago, IL; USA, X-Site; Super Stitch). Suture-mediated vascular closure devices deploy sutures to achieve haemostasis with a knot made either by a built-in device mechanism or manually, which is advanced towards the puncture site to achieve closure of arteriotomy.

The Perclose system (Perclose Inc., Redwood City, CA, USA) introduced in 1994 was the first suture-mediated device to be approved by the United States Food and Drug Administration. Subsequently, various designs were developed such as the Perclose AT, Prostar XL, Closer S, and PP. The PP closure device introduced in 2004 is the latest-generation suture-mediated device from Abbot Laboratories and was approved by the United States Food and Drug Administration in 2013. The PP device offers improvements in the ease of knot delivery; trimming of the suture; and polypropylene monofilaments sutures, which are noninflammatory and characterized by high tensile strength. Each type of device is meant for a particularly sized arteriotomy wound like the X-Site for 6-Fr, Super Stitch for 6-Fr to 8-Fr, Perclose AT for 5-fr to 8-Fr, Prostar for 8-Fr to 10-Fr, and PP for 5-Fr to 21-Fr sheath sizes. For the PP, the recommendation is to use two or more devices in a “preclosure”1 manner if puncture size ≥ 8-Fr.

Due to the use of a larger sheath (up to 26-Fr), the PP closure device effectively provides hemostasis in cases requiring extensive arterial opening. Therefore, it is frequently used in both the TAVI and the EVAR procedures, which have been increasingly used in recent years. Notably, it improves the early mobilization of patients and reduces the length of hospital stay; however, the incidence of PP closure device-related complications was found to be up to 20% in the literature [9, 10]. In a study involving 198 patients who received PP closure devices following the EVAR procedure, the success of the procedure was reported to be 89.9% [11]. However, it was found that there was a complication rate associated with the device of between 10% and 20% in EVAR patients who received the PP closure device [9, 10]. In the present study, three (18.8%) patients who were EVAR at our centre experienced bleeding complications (BARC classes 1, 2, and 3) and two (3.4%) patients undergoing TAVI at our centre demonstrated access site-related bleeding complications. However, in a study conducted involving 387 patients undergoing TAVI by Kiramijyan et al., the minor and major vascular complications were determined to be 18.4% and 8%, respectively, in patients who received the PP closure device [12]. Notably, the mean age in our study was 74.8 years but 82.9 years in the study by Kiramijyan et al. This age difference may be a reason for the lower risk of bleeding and reduced number of vascular complications in our study. When we focus on the main purpose of our study, the incidence of both complications and device failure was higher in the EVAR patients than in the TAVI patients.

Atherosclerotic risk factors play a role in the development of AAA. Especially, hypertension, hyperlipidaemia, and smoking are important factors. In our study, hypertension and hyperlipidaemia were more common in the EVAR group than in the TAVI group. As a result of the effects of these risk factors, intimal atherosclerosis develops, followed by medial hypertrophy, neovascularization, and inflammatory cell infiltration. Beginning with the earliest stages of atherosclerosis development, the distortion of the elastic fibres progresses towards the distal arterial tree through the common iliac arteries as well as the distal abdominal aorta [13, 14]. While the main pathology of TAVI is only on the aortic valve, the entire arterial tree is affected in the pathophysiology of EVAR. Thus, this may be the reason for why the PP closure device has a lower success rate and more vascular complications due to the failure to preload the sutures. The presence of problems with sutures in open surgical procedures reinforces this thesis. In this regard, in a case series of seven patients who underwent open AAA repair, the most common causes of development of endoleaks after surgery were determined to be suture loosening and suture breakage [15]. Patients who underwent femoral cutdown but who did not receive the PP closure device were not included in the study. The number of these patients was 14 in total, and 11 of them underwent EVAR. This suggests that TAVI patients are more appropriate to undergo vascular closure with a percutaneous device.

The steps involved in PP deployment include positioning of the device, needle deployment, suture capture, needle removal, knot advancement, and trimming of the excess thread. Each step requires meticulous care and the chances of technical failure is high if operators are not properly trained. Studies have documented a “learning curve” phenomenon with vascular closure devices. Balzer et al. showed that the learning curve for technical success with suture-based closure was steeper and longer (> 350 patients) [16].

## 5. Limitations

Since our study was designed with a retrospective nature, data on some parameters such as procedure time and fluid intake and output monitoring were missing.

## 6. Conclusion

The use of percutaneous intervention procedures is increasing rapidly, and vascular access site closure significantly affects the mortality and morbidity associated with these procedures. Complications may develop at different rates and types depending on the underlying tissue differences in patients undergoing percutaneous procedures performed for different pathologies. Therefore, macroscopic anatomy as well as conformity to the histopathology of the vascular wall according to aetiology can be an important guide in the evolution of closure devices such as the PP.

## Figures and Tables

**Table 1:** Demographic characteristics of patients undergoing TAVI and EVAR.

**Variables**	**TAVI (N:58)**	**EVAR (N:16)**	**p**
**Age (years)**	76.3±8.3	69.6±6.9	**0.004**

**Sex (male, **%**)**	33 (56.9)	11 (68.8)	0.566

**Hypertension (n,**%**)**	14 (24.1)	9 (56.3)	**0.030**

**Diabetes mellitus (n,**%**)**	2 (12.5)	16 (27.6)	0,327

**CAD (n,**%**)**	14 (87.5)	46 (79.3)	0.720

**Hyperlipidemia (n,**%**)**	19 (32.8)	11 (68.8)	**0.009**

**CVA (n,**%**)**	1 (6.3)	2 (3.4)	0.05

**Heart Failure (n,**%**)**	19 (32.8)	1 (6.3)	0.054

**Antiplatelet (n,**%**)**	9 (25.0)	3 (21.4)	0.791

**Smoking (n,**%**) **	11 (19.3)	6 (40.0)	0.168

**Table 2:** Comparison of laboratory parameters of patients undergoing TAVI and EVAR.

**Variables**	**TAVI (N:58)**	**EVAR (N:16)**	**p**
**Glucose (mg/dL)**	119.1±36.3	101.1±14.2	0.068

**Creatinine (mg/dL)**	1.29 (0.55)	1.45(0.48)	0.844

**TC (mg/dL)**	194.5±61.4	190.5±65.5	0.996

**LDL-C (mg/dL)**	123.4±50.5	128.8±54.1	0.745

**HDL-C (mg/dL)**	43.4±13.4	37.2±8,3	0.127

**Triglyceride (mg/dL)**	136.7±74.4	142.4±53.7	0.798

**AST(mg/dL)**	29.6 (12.2)	24.5 (17.7)	0.577

**ALT(mg/dL)**	25.6 (11)	17.4 (14)	0.669

**GFR (ml/min)**	65.6±26.8	73.3±30.3	0.326

**Hemoglobin(g/dL)**	11.2±1.6	12.1±1.8	0.343

**Hematocrit**	34.1±4.5(%)	35.1±5.2	0.450

**Platelet(**×**10**^**9**^**/L)**	205±75	247±12	0.109

**WBC(mm** ^-**3**^ **)**	8804.3±3602	10003.1±3789	0.248

**ALT:** alanine aminotransferase, **AST:** aspartate aminotransferase, **GFR: **glomerular filtration rate, **HDL:** high density lipoprotein, **LDL:** low density lipoprotein, **EVAR: **endovascular aortic aneurysm repair, **TAVI:** transcatheter aortic valve implantation, **TC:** total cholesterol, and **WBC: **white blood cell count.

**Table 3:** Comparison of results for PP closure device applied in patients undergoing TAVI and EVAR.

**Variables**	**TAVI (N:58)**	**EVAR (N:16)**	**p**
**Complication (n,**%**)**	2 (3.4)	3 (18.8)	**0.031**

**Device failure (n,**%**)**	2 (3.4)	3 (18.8)	**0.031**

**Number of PP closure deviceused**	2.3±0.6	4.5±1.4	<**0.001**

**Right CFA diameter (mm)**	8.6±1.5	7.7±1.4	0.186

**Left CFA diameter (mm)**	8.8±1.5	7.8±1.2	0.172

**CFA: **common femoral artery, **EVAR:** endovascular aortic aneurysm repair, and **TAVI:** transcatheter aortic valve implantation.

## Data Availability

The data used to support the findings of this study are available from the corresponding author upon request.
